# Selenoprotein P Regulates Synaptic Zinc and Reduces Tau Phosphorylation

**DOI:** 10.3389/fnut.2021.683154

**Published:** 2021-07-01

**Authors:** Arlene C. P. Kiyohara, Daniel J. Torres, Ayaka Hagiwara, Jenna Pak, Rachel H. L. H. Rueli, C. William R. Shuttleworth, Frederick P. Bellinger

**Affiliations:** ^1^Department of Cell and Molecular Biology, John A. Burns School of Medicine, University of Hawaii at Manoa, Honolulu, HI, United States; ^2^Pacific Biosciences Research Center, School of Ocean and Earth Science and Technology, University of Hawaii at Manoa, Honolulu, HI, United States; ^3^Department of Neurosciences, University of New Mexico, Albuquerque, NM, United States

**Keywords:** Selenoprotein P, zinc, Alzheimer's disease, tau, selenium

## Abstract

Selenoprotein P (SELENOP1) is a selenium-rich antioxidant protein involved in extracellular transport of selenium (Se). SELENOP1 also has metal binding properties. The trace element Zinc (Zn^2+^) is a neuromodulator that can be released from synaptic terminals in the brain, primarily from a subset of glutamatergic terminals. Both Zn^2+^ and Se are necessary for normal brain function. Although these ions can bind together with high affinity, the biological significance of an interaction of SELENOP1 with Zn^2+^ has not been investigated. We examined changes in brain Zn^2+^ in SELENOP1 knockout (KO) animals. Timm-Danscher and N-(6-methoxy-8-quinolyl)-*p-*toluenesulphonamide (TSQ) staining revealed increased levels of intracellular Zn^2+^ in the SELENOP1^−/−^ hippocampus compared to wildtype (WT) mice. Mass spectrometry analysis of frozen whole brain samples demonstrated that total Zn^2+^ was not increased in the SELENOP1^−/−^ mice, suggesting only local changes in Zn^2+^ distribution. Unexpectedly, live Zn^2+^ imaging of hippocampal slices with a selective extracellular fluorescent Zn^2+^ indicator (FluoZin-3) showed that SELENOP1^−/−^ mice have impaired Zn^2+^ release in response to KCl-induced neuron depolarization. The zinc/metal storage protein metallothionein 3 (MT-3) was increased in SELENOP1^−/−^ hippocampus relative to wildtype, possibly in response to an elevated Zn^2+^ content. We found that depriving cultured cells of selenium resulted in increased intracellular Zn^2+^, as did inhibition of selenoprotein GPX4 but not GPX1, suggesting the increased Zn^2+^ in SELENOP1^−/−^ mice is due to a downregulation of antioxidant selenoproteins and subsequent release of Zn^2+^ from intracellular stores. Surprisingly, we found increased tau phosphorylation in the hippocampus of SELENOP1^−/−^ mice, possibly resulting from intracellular zinc changes. Our findings reveal important roles for SELENOP1 in the maintenance of synaptic Zn^2+^ physiology and preventing tau hyperphosphorylation.

## Introduction

Within the body, selenium (Se) functions primarily in the form of selenocysteine (Sec), the 21st amino acid, which is incorporated into members of the selenoprotein family (1–3). Selenoprotein P (SELENOP1) is a selenium-rich protein with 10 Sec residues that transports Se in serum from liver to the brain and other organs (4). SELENOP1 is present in the cerebral spinal fluid (CSF) and in the choroid plexus, which releases CSF (5, 6), and in glial cells (7). SELENOP1 has also been described in brain neurons (8, 9), which may be the targets of Se transport. SELENOP1 KO mice have reduced brain selenium and reduced levels of antioxidant selenoproteins such as glutathione peroxidases 1 and 4 (GPX1 and GPX4) (10). Mice with the SELENOP1 gene deletion have deficient hippocampal synaptic function and deficits in spatial learning and long-term potentiation (LTP), a model for learning and memory (11). SELENOP1 is increased in the brain and CSF in Alzheimer's disease (5, 8, 12) and associated with both Alzheimer's and Parkinson's pathology (8, 13).

Se and Zn^2+^ are both essential trace elements required for proper brain function. Selenium deficiency correlates with impaired cognitive and motor function (14, 15), while Zn^2+^ deficiency correlates with decreased nerve conduction and impaired cognitive performance (16). Alzheimer's disease is associated with increased brain Zn^2+^ levels (17). Zn^2+^ can increase tau phosphorylation (18, 19), which contributes to the formation of neurofibrillary tangles, a hallmark of Alzheimer's disease (20). However, studies have yet to address the biological relevance of the interaction between these elements despite their high affinity for each other and their importance in brain function.

**Figure 1:**
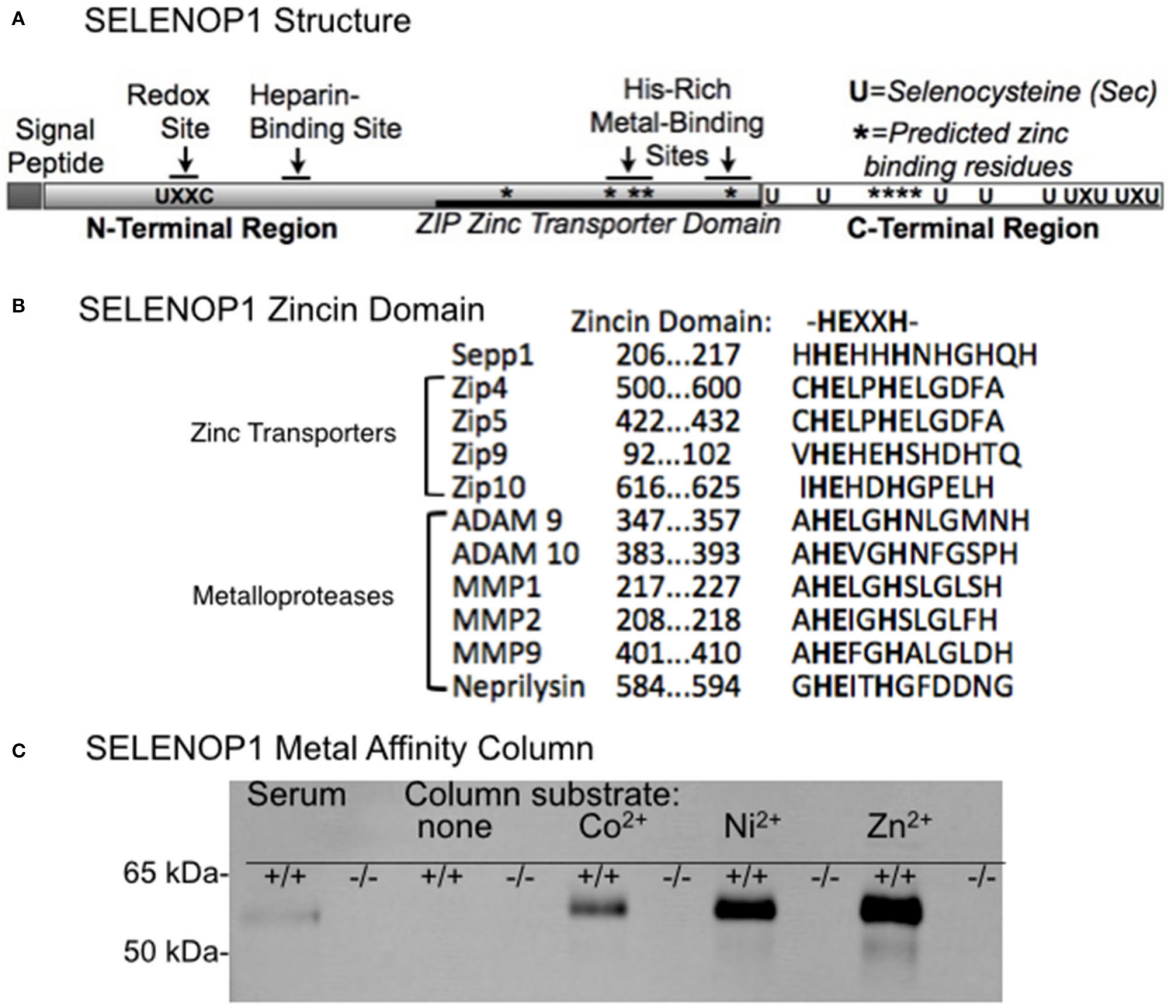
Zinc-binding properties of SELENOP1. **(A)** Schematic of SELENOP1 structure. The N-terminal region has 1 S (U) in a redox domain, a heparin-binding site and a zinc transporter domain with two metal-binding sites. The C-terminal region has 9 S residues for Se transport. Sites of residues predicted to bind zinc using Predzinc are indicated by *. **(B)** Alignment of human SELENOP1 metal binding region with other proteins containing the zincin motif. **(C)** Western blot of metal column eluates after applying mouse serum from WT (+/+) or SELENOP1 KO (^−/−^) mice to mini columns with agarose only or agarose bound to Co^2+^, Ni^2+^, and Zn^2+^. Untreated SELENOP1^−/−^ and WT serum were also added to the blot as a positive control (left lanes). SELENOP1 protein detected with anti-SELENOP1 antibody (1:1000) had a molecular weight of approximately 55 kDa in the wildtype serum, which was not seen in SELENOP1^−/−^ serum. SELENOP1 was detected in column eluates from wild-type serum applied to all metal columns, but not from columns with agarose only.

Figure 1A shows SELENOP1's two functional, glycosylated domains: (1) a Se-rich C-terminal domain with 9 S residues, (2) an N-terminal domain with 1 S (U) in U-x-x-C redox motif, 2 histidine-rich metal binding sites (located at residue 204–217 and residue 244–250) and a heparin binding site (4, 21). SELENOP1 also has an N-terminal signal peptide for extracellular secretion, which is cleaved in the Golgi (22).

We hypothesized that SELENOP1, as a metal-binding protein, could have a role in brain Zn^2+^ homeostasis. In this study, we investigated whether SELENOP1 influences brain Zn^2+^ by evaluating changes in hippocampal Zn^2+^ in SELENOP1 KO animals. Here we report that deletion of SELENOP1 alters levels of chelatable Zn^2+^ and prevents the release of synaptic Zn^2+^ in mouse hippocampus. These findings indicate an important additional role for SELENOP1 in the regulation of zinc in the brain. They could also have important implications for the treatment of disorders where Zn^2+^ physiology is impaired.

## Materials and Methods

### Animals

Mice were group housed on a 12-h light cycle and provided food and water *ad libitum*. All animals in this study were maintained on diets containing adequate selenium (~0.25 ppm) and zinc (~80 ppm). All mice used were 3–6 months of age and included both male and female mice as indicated. All animal procedures were approved by the University of Hawaii Institutional Animal Care and Use Committee.

SELENOP1^−/−^ mice were obtained from the laboratory of Dr. Raymond Burk at Vanderbilt University. The mutant mice were backcrossed to C57BL/6J for at least ten generations with C57BL/6J mice from Jackson Laboratories to ensure congenic strains. Breeding of SELENOP1^+/−^ mice generated littermates of SELENOP1^+/+^ and SELENOP1^−/−^ pups, which were used in this study in addition to SELENOP1^+/−^ mice. Genomic DNA extracted from mice tails was used for genotyping PCR using specific primers (forward-ACCTCAGCAATGTGGAGAAGCC, reverse-TGCCCTCTGAGTTTAGCATTG for wild-type, and reverse-GATGATCTGGACGAAGAGCATCA for SELENOP1^−/−^). Products were run on a 1.5% DNA agarose gel with a SYBR Safe DNA gel stain (Invitrogen) and genotypes were confirmed under UV light.

### Timm-Danscher Zn^2+^ Labeling

The Danscher modification of Timm's zinc stain (“neo-Timm's”) was used to label intracellular chelatable zinc (i.e., not tightly bound to proteins or other molecules) (23). Deeply anesthetized mice received intraperitoneal (IP) injections of 20 mg/kg sodium selenite (2 mg/ml in normal saline). After 2 h, mice were perfused with saline followed by 4% paraformaldehyde (PFA) in saline. Brains were postfixed overnight in 4% PFA, dehydrated serially in 10% and 30% sucrose, and mounted in optimal cutting temperature compound (OCT) for cryostat sectioning. After thoroughly washing in PBS, sections were developed with the IntenSE M silver Enhancement kit (Amersham International) according to the manufacturer's protocol and as previously described (24).

### 6-Methoxy-8-P-Toluenesulfonamido-Quinoline (TSQ) Stain

TSQ is a Zn^2+^ fluorophore that binds intracellular chelatable Zn^2+^ in a 2:1 ligand-to-metal ratio that results in increased fluorescence emission at 490 nm in response to excitation at 360 nm (25). Serial sagittal cryosections (10 μm) of brain hemispheres were mounted on positively charged microscope slides. The slides were immersed with 4.5 μM TSQ (Enzo Lifesciences, UltraPure) in 140 mM sodium barbital and 140 mM sodium acetate buffer (pH 10) for 90 s, as previously described (26) and washed in 0.1% NaCl. TSQ-stained sections were imaged using DAPI filter settings (200 ms, monochrome with a 5x objective). The mean fluorescence intensity of the hippocampal CA1 stratum oriens and stratum radiatum, CA3 mossy fibers, and hilar region were measured with ImageJ software (NIH). The background was measured in unstained areas within lateral ventricles and subtracted from mean TSQ signals.

### Inductively Coupled Plasma Optical Emission Spectroscopy (ICP-OES)

The metal concentrations within frozen right brain hemispheres and liver samples were processed at the Agricultural Diagnostic Service Center (ADSC) run by the College of Tropical Agriculture and Human Resources (CTAHR), University of Hawaii. Dry ash sample preparations were subjected to acid digest before ICP-OES (0.01 ppm detection limit) to measure total brain and liver metal content (Zn^2+^, Cu^2+^, Fe^2+^). Water blanks and solution standards were included with each run to calibrate results.

### Protein Extraction and Western Blot

Proteins were extracted from frozen hippocampal tissue using CelLytic MT buffer (Sigma) per the manufacturer's instructions, denatured by heating in Laemmli sample buffer, resolved by SDS-PAGE on a 10–20% gradient Tris-HCl Criterion Precast gel (Bio-Rad Laboratories), and electrically transferred to polyvinylidene difluoride (PVDF) membranes. For detection of Zn^2+^ regulating proteins, membranes were incubated in anti-metallothionein-3 (1:500, rabbit polyclonal, Biorbyt), and anti-ZnT1 and anti-ZnT3 (1:1000; 1:5000, rabbit polyclonal, Synaptic Systems). For detection of the SELENOP1 protein, membranes were incubated in anti-SELENOP1 (1:1000, rabbit monoclonal, Proteintech). For measurement of tau, antibodies recognizing tau phosphorylated at T231, S214 or S396 (1:1000, Invitrogen) or tau 5 (1:1000, Millipore). Membranes washed and then treated with corresponding secondary antibodies conjugated with infrared fluorophores (1:10,000; Licor). Blots were subsequently treated with anti α-tubulin (1:5000; Novus Biologicals) to control for loading. Membranes were imaged with the Odyssey infrared fluorescence system (LiCor), and densitometry analysis was performed on the Imagestudio software (LiCor).

### Metal Agarose Column Purification

To observe SELENOP1 binding to biometals, we used mini spin-columns containing high-density agarose beads conjugated with Zn^2+^, Ni^2+^, and Co^2+^ (Agarose Bead Technologies) to isolate metal-binding proteins from wildtype and SELENOP1^−/−^ mouse serum. A metal-free agarose column served as a negative control. Serum samples were diluted 1:100 in PBS and added to the column, gently shaken for 60 min at 4°C, then spun for 60 s at 800x g to collect flow-through. Columns were washed with increasing concentrations of imidazole (0, 10, and 20 mM) diluted in PBS, and then bound proteins were eluted with 250 mM imidazole diluted in PBS. Eluted proteins were determined with western blot using an anti-SELENOP1 antibody (27).

### Live Hippocampal Slice Imaging

To measure stimulus-induced extracellular Zn^2+^accumulation, hippocampal slices were prepared from 3 to 6 month old SELENOP1 KO and wild-type littermate mice as previously described (28). Following slice preparation, slices were acclimated to room temperature and superfused with oxygenated (95 O_2_ and 5% CO_2_ gas mix) artificial cerebral spinal fluid (ACSF, composition in mM: NaCl 130; KCl 3.5; glucose 10; NaHCO_3_ 24; NaH_2_PO_4_ 1.25; MgSO_4_ 1.5; CaCl_2_ 2.0) for at least 60 min. The CA1 stratum radiatum region of the slices was imaged with a Zeiss laser-scanning microscope using a 10X objective with the pinhole fully opened at 1 frame/s at 640 × 480 resolution. Cell-impermeant FluoZin-3 at 1.5 μM (Molecular Probes) was added to the ACSF to detect extracellular Zn^2+^ accumulation from hippocampal slices in response to the administration of a depolarizing (35 mM) KCl concentration for 60 s. In some experiments, a slow onset Zn^2+^ chelator (Ca^2+^-EDTA) was added to remove contaminating Zn^2+^ in media and to reduce background fluorescence (29, 30). Fluorescence intensities of hippocampal slices in the CA1 stratum radiatum region upon addition of KCl for each slice were expressed as the fluorescence intensity over the fluorescence during baseline (F/F_0_). The mean area under the curve during application of high K^+^ for signals of SELENOP1^−/−^ slices were compared to WT to determine if changes in Zn^2+^ release were altered.

### FluoZin-3 Measurements in Cell Culture

SH-SY5Y cells were plated in 96-well plates and differentiated by exposure to Neurobasal media (Invitrogen) supplemented with B27 (Invitrogen) for 48 hrs. The media was then changed to Roth-Schweizer media (31) but with of 0, 10 or 100 nM. Alternatively, cultures in 10 nM Se were treated with 100 μM mercaptosuccinate (MCS), 0.1 μM RSL-3, 0.1 μM RSL-3 + 100 μM α-tocopherol, or 0.01 μM DMSO as a control for RSL-3. Cells were treated for either 5 days (DMSO, RSL-3, RSL-3 with α-tocopherol) or 7 days (Se concentrations and remaining conditions). Cells were rinsed twice in HEPES buffered saline [HBSS: (in mM) NaCl 146; KCl 3.5; glucose 10; 1.25; MgSO_4_ 1.5; CaCl_2_ 2.0, HEPES 10, NaOH 10]. Cells were loaded with 1 μM FluoZin-3 AM (Thermo Fisher Scientific) with 0.02% pluoronic F-127 for 30 min at 37°C in the dark, then rinsed twice with HBSS, and the fluorescence measured in HBSS. For additional controls, either 100 μM 2, 2′-dithiodipyridine (DTDP), 100 μM tetrakis-(2-pyridylmethyl) ethylenediamine (TPEN), or 100 μM H_2_O_2_ were added to the HBSS 10 min before measuring fluorescence. Plates were scanned in a SpectraMax M3 fluorescent plate reader (Molecular Devices) with 494 nm excitation, 516 nm emission. After scanning, cells were fixed in 100% methanol at −20°C overnight, stained with 100 μg propidium iodide (PI), rinsed twice with HBSS, than scanned in HBSS at 536 nm excitation, 617 nm emission. FluoZin-3 fluorescence was normalized to PI fluorescence to correct for any differences in cell density.

### Statistical Analysis

All statistical analyses were carried out with Graphpad Prism Software with measurements given as means ± SE. Comparisons between treatments, genotypes and sex were performed by student's unpaired *t*-test and one-way or two-way analysis of variance (ANOVA), with *p* < 0.05 considered significant. In general, we did not find sex differences in Zn^2+^ distribution or release unless shown; otherwise, males and females were averaged together by genotype.

## Results

### Zn^2+^ Binding Properties of SELENOP1

Previous studies described the affinity of SELENOP1 for several metals, including Zn^2+^. However, the biological interaction of SELENOP1 with Zn^2+^ is unclear. We investigated potential Zn^2+^-binding domains of SELENOP1. Using the web-based software Predzinc (https://predzinc.bioshu.se/pred/), a web server that predicts zinc-binding proteins and zinc-binding sites from given sequences, we analyzed the SELENOP1 coding sequences for potential zinc-binding sites. We found that a His-residue within the SELENOP1 His-rich metal binding domain as well as two other His residues in the C-terminal region are predicted to be Zn^2+^ binding motifs, shown in Figure 1A by asterisks (^*^).

A domain search of the Kyoto Encyclopedia of Genes and Genomes (KEGG) database indicates that the human SELENOP1 protein structure has a zinc-binding domain overlapping one of the His-rich regions, which has homology to the ZIP zinc transporter domain. Additionally, within this sequence is a zincin Zn^2+^-binding motif, generally found in the metzincin family of metalloproteases (Figure 1B) (32).

Because of the predicted Zn^2+^-binding region of SELENOP1, we tested SELENOP1's potential to bind Zn^2+^ ions by passing mouse serum through agarose columns bound to Co^2+^, Ni^2+^, or Zn^2+^, or metal-free as a negative control. Western blot of sample elution from each column purification shows that SELENOP1 binds to Co^2+^, Ni^2+^, and Zn^2+^, but not to the metal-free agarose column (Figure 1C). When serum from SELENOP1^−/−^ mice was used, SELENOP1 immunoreactivity was not detected in eluent from any of the columns. This demonstrates that SELENOP1 is capable of binding different biometals, with a greater amount of binding to Zn^2+^ compared with Co^2+^ and Ni^2+^.

### Elevated Levels of Intracellular Zn^2+^ in SELENOP1^–/–^ Hippocampus

**Figure 2:**
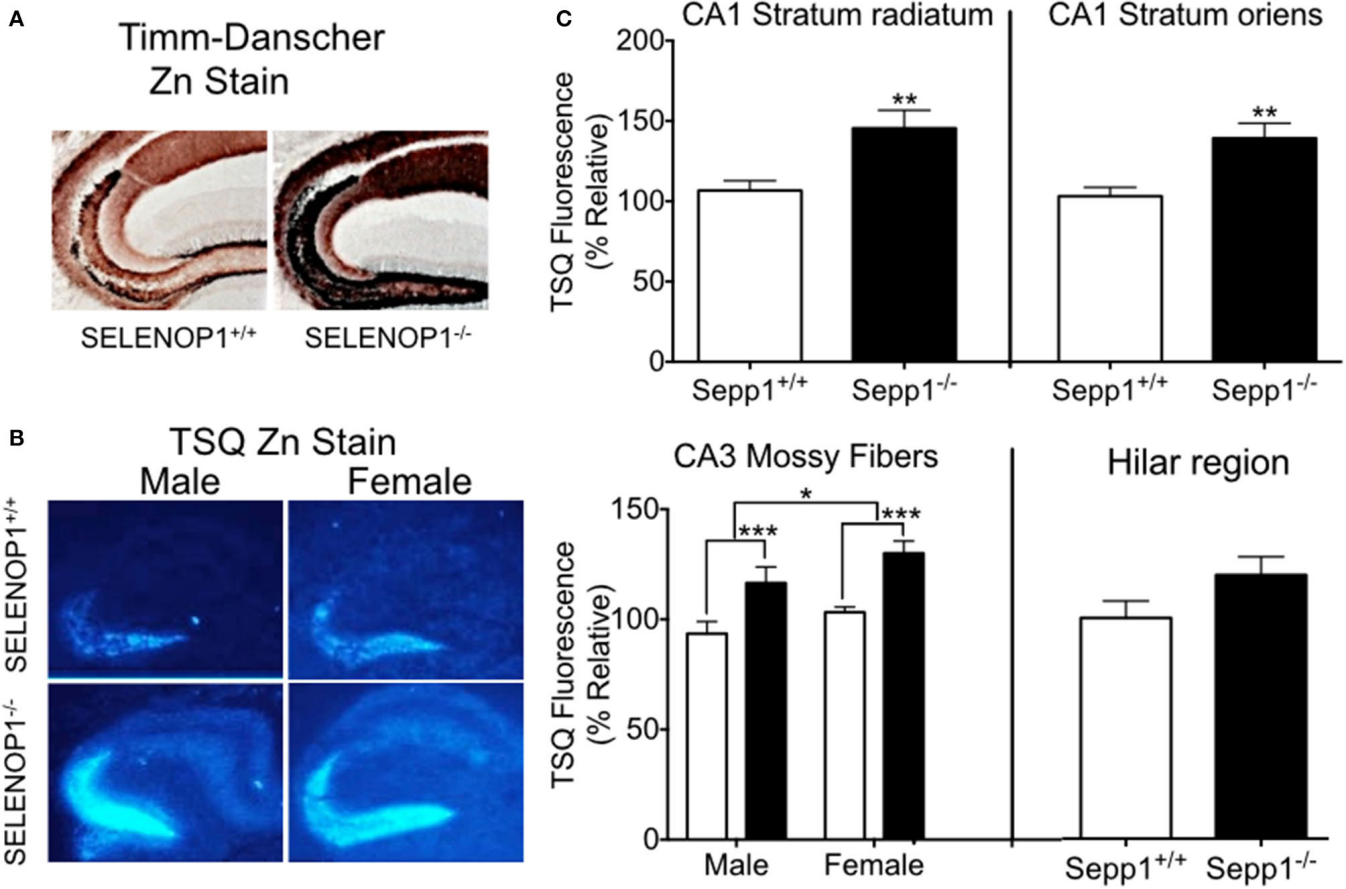
Increased hippocampal zinc in SELENOP1 KO mice. **(A)** Neo-Timm's (Danscher) stain. Free Zn^2+^ in wildtype and SELENOP1^−/−^ hippocampal neurons from mice injected with sodium selenite is revealed as a reddish-brown to dark brown color relative to the amount of total Zn^2+^. **(B)** Representative images from male and female WT and SELENOP1^−/−^ mouse hippocampus, labeled with the fluorescent Zn^2+^ indicator, TSQ. **(C)** Quantitation of TSQ fluorescent label indicates higher Zn^2+^ levels in SELENOP1^−/−^ animals in all observed regions except for hilar region. The average values for each region were normalized to wild type males. *N* = 6 animals/group, 5–10 slices/animal. Statistical analysis is performed by Student's unpaired t- test or two-way ANOVA with Bonferroni's *post-hoc* test. *p < 0.05, **p < 0.01, ***p < 0.001.

To explore the effects of SELENOP1 on brain Zn^2+^ homeostasis, we first evaluated intracellular Zn^2+^ levels in SELENOP1^−/−^ mice using histological methods. Our studies focused on the hippocampus as a zinc-rich region important for learning and memory (33). The Timm-Danscher method (34) revealed a pronounced increase in histologically-detectable Zn^2+^ in the hippocampus of SELENOP1^−/−^ compared with SELENOP1^+/+^ hippocampus (Figure 2A). The hilar and CA3 mossy fiber regions had the most intense Zn^2+^ labeling in both wild-type and SELENOP1^−/−^ animals. However, all layers positive for Zn^2+^ were increased in KOs, including the stratum radiatum and stratum oriens of the CA1 and CA3 layers.

We further compared bioreactive Zn^2+^ levels in SELENOP1^−/−^ and wildtype mouse hippocampi using TSQ labeling. Quantitation of TSQ fluorescence also revealed significantly higher levels of intracellular Zn^2+^ in the CA1 stratum oriens, stratum radiatum, and CA3 mossy fibers of the SELENOP1^−/−^ animals compared to their control (Figures 2B,C). We also observed a sex difference in CA3, with increased Zn^2+^ in mossy fibers in female compared to male hippocampus regardless of genotype. Unstained hippocampal brain sections showed no fluorescence at TSQ wavelengths, indicating that differences were not due to autofluorescence signals. Hematoxylin staining of sections previously used for TSQ labeling showed no morphological differences between SELENOP1 wildtype and KO hippocampi. Based on our findings, SELENOP1 may be regulating Zn^2+^ levels directly or through one or more Zn^2+^ interacting proteins.

As we observed increased levels of intracellular Zn^2+^ within the hippocampus of SELENOP1^−/−^ mice, we then investigated if the total brain Zn^2+^ levels are altered by subjecting whole brain hemispheres via ICP-OES to measure total metal content for Zn^2+^, Cu^2+^, and Fe^2+^. There was no significant increase in total brain Zn^2+^ levels in SELENOP1^−/−^ mice (Table 1). This may indicate that deletion of SELENOP1 results in changes to the distribution of Zn^2+^ within the brain rather than an increase in total brain Zn^2+^.

**Table 1:** Metal content in brain and liver of Sepp1 wildtype (Sepp1^+/+^) and knockout (Sepp1^−/−^) mice at the age of 3 months (mg/kg of dry tissue, *n* = 20 each) measured by ICP-OES.

**Tissue**	**Metal**	**Genotype**		*P*-value^*^
		**Sepp1^+/+^**	**Sepp1^−/−^**	
Brain	Zn	12.04 ± 0.23^a^	12.34 ± 0.32	0.45
	Cu	4.480 ± 0.16	4.383 ± 0.29	0.77
	Fe	19.99 ± 1.31	18.25 ± 1.04	0.30
Liver	Zn	25.85 ± 1.04	25.20 ± 1.48	0.72
	Cu	6.048 ± 0.42	6.756 ± 0.61	0.35
	Fe	88.26 ± 4.34	90.91 ± 7.85	0.77

* *Unpaired two-tailed t-test was used to determine the probability of differences between genotypes*.

a *Mean ± SEM*.

### Expression Levels of Zn^2+^-Interacting Proteins

**Figure 3:**
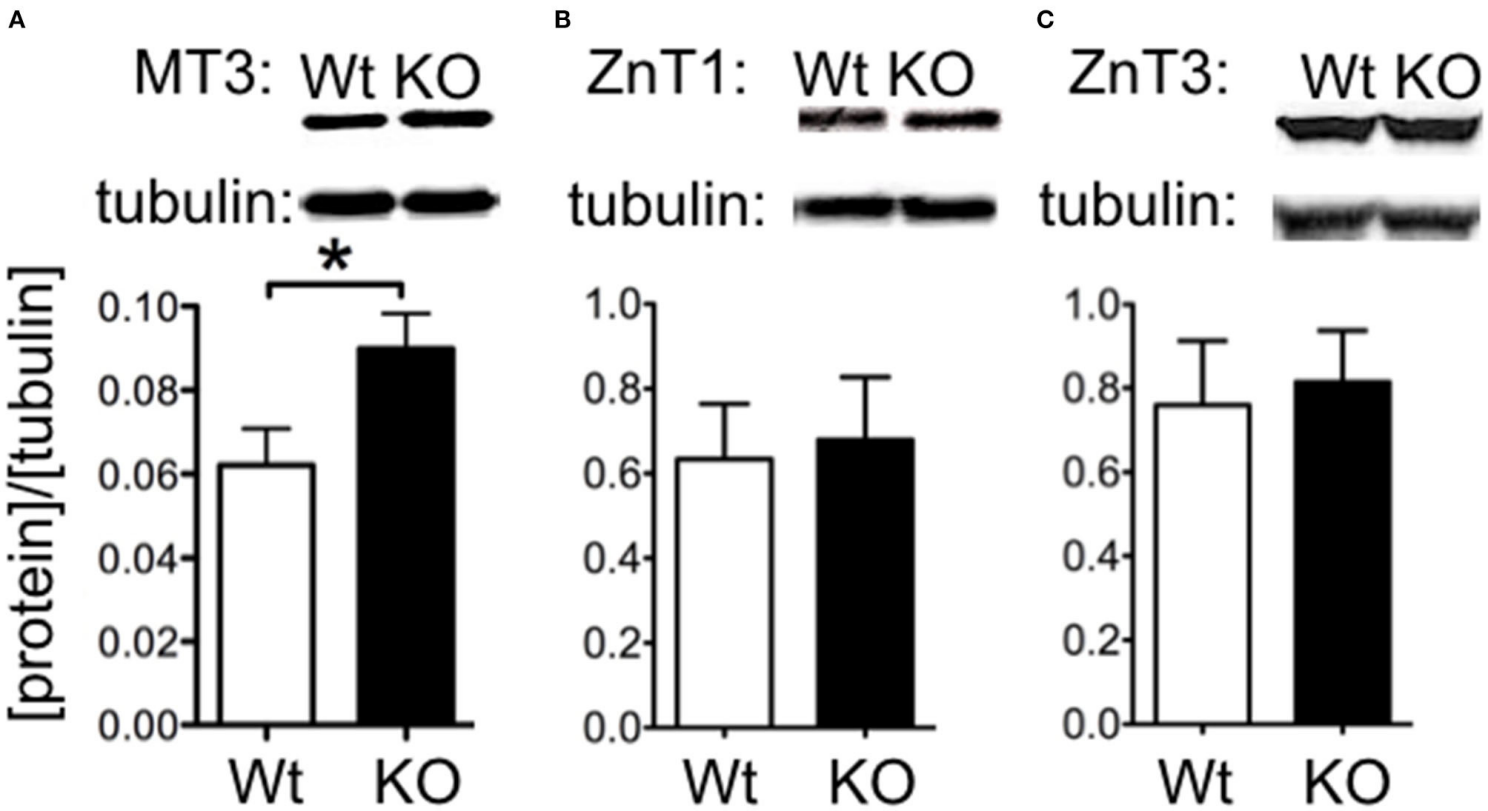
Increased MT3 but not ZnT1 or ZnT3 protein levels in SELENOP1 KO hippocampus. Western blot showing expression levels of Zn^2+^ transporter proteins, ZnT1 and ZnT3, and Zn^2+^ storage pool, MT3. Protein densities were normalized to α-tubulin and averaged to male WT values. **(A)** ZnT1 protein levels were unchanged. **(B)** ZnT3 protein was also unchanged. **(C)** Increased MT3 expression. Representative bands are shown above each corresponding bar graph and were found to run at the predicted sizes: ZnT1 (45kDa), ZnT3 (42kDa), and MT3 (40kDa). Statistical analysis is performed by Student's unpaired *t*-test, *p < 0.05.

We investigated whether changes in Zn^2+^ regulating proteins could explain differences in Zn^2+^. We investigated hippocampal expression of the Zn^2+^ transporters ZnT1 and the vesicle-associated ZnT3, as well as the metal storage protein MT3. Western blot indicated that ZnT1 and ZnT3 proteins were unchanged in SELENOP1^−/−^ animals (Figures 3A,B). However, MT3 protein expression was increased (Figure 3C), suggesting that deletion of the SELENOP1 gene does not affect zinc transport through these major pathways, but rather upregulates expression of the Zn^2+^ storage protein. The enhanced expression of MT3 may be a result of a feedback mechanism in response to increased Zn^2+^ levels in the SELENOP1^−/−^ hippocampus.

### Zn^2+^ Release Is Impaired in SELENOP1^–/–^ Hippocampus in Response to Neuron Depolarization

**Figure 4:**
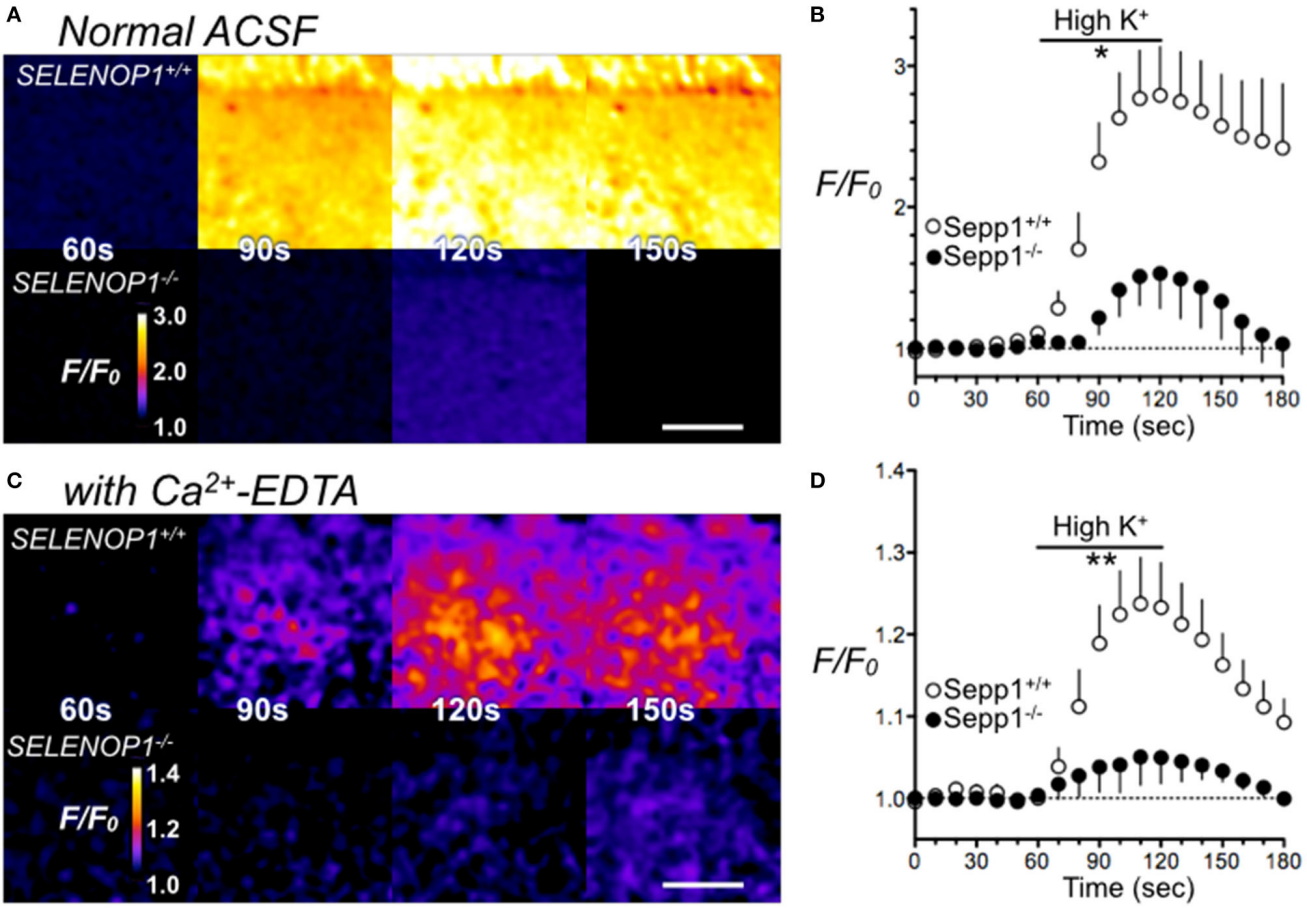
Impaired Zn^2+^ release in SELENOP1 KO hippocampus. **(A)** Zn^2+^ release from hippocampal slices of SELENOP1^−/−^ and WT mice visualized with Fluozin-3 following depolarization induced with ACSF containing high (35 mM) KCl solution. Fluorescence in SELENOP1^−/−^ hippocampal slices was significantly reduced compared to WT animals. **(A)** Representative Fluozin-3 images from WT and SELENOP1^−/−^ animals at 60s, 90s, 120s and 150s, after adding high K^+^ from 60 to 120 s. **(B)** Data plotted as relative fluorescence (F/F_0_) over time with measurements averaged every 10 s. WT, *n* = 6 slices, 3 mice; SELENOP1^−/−^, *n* = 5 slices, 3 mice. **(C)** Zn^2+^ release visualized with Fluozin-3 in ACSF with the addition of Zn^2+^ chelator, Ca^2+^-EDTA to reduce background fluorescence, following application of ACSF with high (35 mM) KCl. Representative Fluozin-3 images from WT and SELENOP1^−/−^ animals at 60, 90, 120, and 150 s, after adding high K^+^ from 60 to 120 s. **(D)** Data plotted as relative fluorescence (F/F_0_) over time with measurements averaged every 5 s. WT, *n* = 3 slices, 3 mice; SELENOP1^−/−^, *n* = 4 slices, 4 mice; Fluorescence in SELENOP1^−/−^ hippocampal slices was significantly reduced compared to WT animals. Statistical analysis was performed by Student's unpaired *t*-test comparing the mean area under the curve for each genotype between 60 and 120 s, *p < 0.05, **p < 0.01.

Most histologically-detectable Zn^2+^ in hippocampal neurons is vesicular (25). We investigated if the increased Zn^2+^ levels visualized by TSQ or Timm's staining in SELENOP1^−/−^ mice results in increased extracellular accumulation following depolarizing stimuli designed to promote vesicular release. We imaged extracellular Zn^2+^ accumulation in hippocampal slices with a selective cell-impermeant fluorescent Zn^2+^ indicator, Fluozin-3, in the extracellular media. By depolarizing hippocampal cells with the addition of 35 mM KCl, we noticed an increase in fluorescence in slices from wild type mice, indicating release of Zn^2+^ into the extracellular space (Figures 4A,B). In contrast, we observed a minimal increase in fluorescence in hippocampal slices from SELENOP1^−/−^ mice, indicating negligible Zn^2+^ release in the SELENOP1^−/−^ hippocampus.

A high background fluorescence recording from Zn^2+^ contaminants in the reagents used could possibly mask Zn^2+^ release. To reduce background fluorescence, we also imaged slices with the Zn^2+^ chelator, Ca^2+^-EDTA, added to the ACSF. Ca^2+^-EDTA enables removal of basal levels of extracellular Zn^2+^, but its slow kinetics do not prevent the detection of synaptically-evoked Zn^2+^ accumulation (35). Even in the presence of Ca^2+^-EDTA, we still observed significantly larger FluoZin-3 increases in wild-type hippocampus slices relative to SELENOP1^−/−^ slices upon cell depolarization (Figures 4C,D). The baseline fluorescence (F_0_) was not significantly different between SELENOP1^+/+^ and SELENOP1^−/−^ slices in either normal ACSF for Ca^2+^-EDTA ACSF.

### Selenium Deficiency Releases Intracellular Zn^2+^

**Figure 5:**
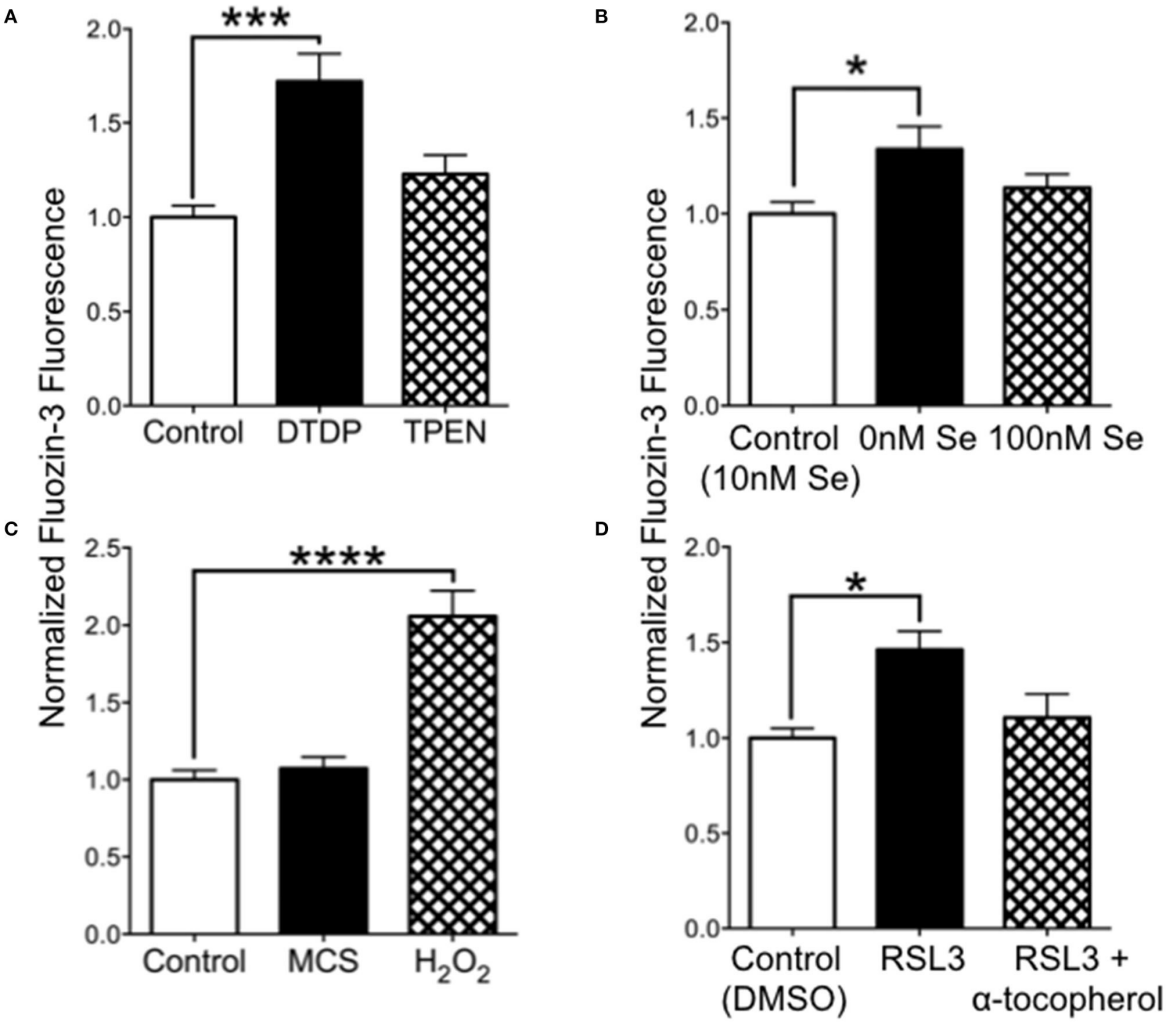
Intracellular Zn^2+^levels are increased by 0 Se and GPX4 inhibitor RSL-3. **(A)** Relative FluoZin-3 fluorescence of SH-SY5Y cells. Cells exposed to DTDP to release zinc showed increased fluorescence, while cells exposed to PTEN to reduce intracellular zinc had no change in fluorescence. **(B)** FluoZin-3 fluorescence was increased in cells grown in media with 0 nM Se relative to control media (10 nM), while 100 nM Se did not alter intracellular zinc. **(C)** Hydrogen peroxide but not GPX1 inhibitor mercaptosuccinate (MCS) increase intracellular zinc. **(D)** The GPX4 inhibitor RSL-3 increased intracellular zinc, which was reversed when RSL-3 was added with α-tocopherol. Statistical analysis is performed by one-way ANOVA followed by Dunnett's multiple comparison test. *p < 0.05, ***p < 0.005, ****p < 0.0001.

We hypothesized that increased oxidation from Se-deficient conditions could result in reduced intracellular chelation of Zn^2+^. We tested this by measuring the fluorescence of cell-permeable FluoZin-3 in cultured SH-SY5Y cells. As shown in Figure 5A, release of intracellular zinc by DTDP, a thiol oxidizer which liberates intracellular Zn^2+^, significantly increased FluoZin-3 fluorescence. However, the intracellular Zn^2+^ chelator TPEN did not reduce FluoZin-3 fluorescence, indicating that free non-chelated Zn^2+^ levels were too low to be detectable. We also found that cells grown in 0 Se culture media had significantly increased fluorescence compared with cells grown in our baseline media with 10 nM Se (Figure 5B). Oxidation with H_2_O_2_ greatly increased FluoZin-3 fluorescence, demonstrating that oxidation could free Zn^2+^ from stores. To test if a reduction in activity of the H2O2-reducing selenoprotein GPX1, which depends on Se for synthesis, could lead to increased intracellular Zn^2+^, we exposed cells to the GPX1 inhibitor mercaptosuccinate (MCS) (Figure 5C). MCS did not alter fluorescence, however, we found that RSL-3, an inhibitor of the phospholipid peroxidase selenoprotein GPX4, increased intracellular Zn^2+^ (Figure 5D). This increase was prevented by the vitamin E compound α-tocopherol, which reduces lipid peroxidation. These findings suggest that a deficiency in Se can increase free intracellular Zn^2+^ by causing a reduction in GPX4, which results in increased oxidation of lipids.

### Elevated Tau Phosphorylation in SELENOP1 Knockout Hippocampus

**Figure 6:**
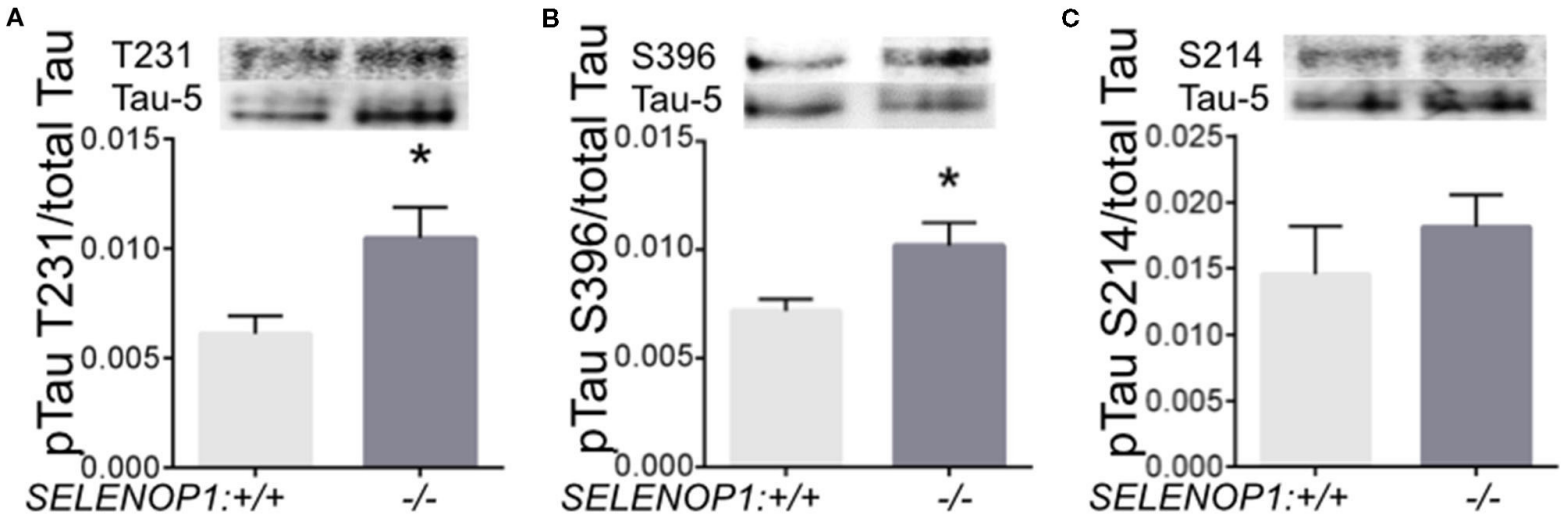
Deletion of SELENOP1 increases tau phosphorylation in mouse hippocampus. Western blots of phospho-specific tau antibodies relative to the Tau-5 antibody, which recognizes total tau. Blot images are superimposed above graphs. **(A)** SELENOP1 deletion resulted in increased tau phosphorylation at threonine 231. **(B)** Serine 396 was also significantly reduced in SELENOP1 knockouts. **(C)** No change in phosphorylation at serine 214. *N* = 4 animals/group. Statistical analysis is performed by Student's unpaired *t*-test, *p < 0.05.

Zn^2+^ promotes tau phosphorylation leading to neurofibrillary tangle formation (18). We questioned whether tau phosphorylation could be altered in the SELENOP1^−/−^ mouse. We performed western blot analysis to compare specific pTau sites to total tau protein. We found that phosphorylation at threonine 231 and at serine 396 were significantly increased in SELENOP1^−/−^ mice (Figure 6A). However, phosphorylation at the serine 214 site was unchanged (Figures 6B,C). Deletion of SELENOP1^−/−^ thus results in a site-specific increase in tau phosphorylation.

## Discussion

Our findings show that deletion of SELENOP1 increased free (chelatable) intracellular Zn^2+^ levels. However, release of synaptic zinc was impaired. Although the Zn^2+^ storage protein MT3 was elevated without change to the zinc transporters ZnT1 or ZnT3, we showed that selenium deficiency could induce release of Zn^2+^ from stores, likely through a decrease of the selenoprotein GPX4 and a subsequent increase in lipid peroxidation. Interestingly, GPX4 helps to prevent neurodegeneration though ferroptosis (36). Lastly, we demonstrated an increase in site-specific phosphorylation of tau. These findings suggest that SELENOP1 plays a role in regulating storage of intracellular Zn^2+^. This role may be important for preventing tau hyperphosphorylation in AD.

Dietary supplementation with selenium in the form of sodium selenate reduces tau phosphorylation to potentially reduce neurofibrillary tangle formation (37, 38). Selenate can act as an agonist for protein phosphatase 2A (PP2A), which targets tau phosphorylation (39). Interestingly, Zn^2+^ is an inhibitor of PP2A, and may promote neurofibrillary tangle formation (18). Hyper-phosphorylation of tau leads to neurofibrillary tangle formation, and de-phosphorylation by PP2A should reduce tangle formation. However, dietary selenate supplementation also upregulates the expression of selenoproteins (40, 41). We have previously reported that a reduction in selenoprotein S can promote tau phosphorylation (42). In a type 2 early clinical trial for selenate supplementation in Alzheimer's disease, selenate could increase brain selenium in some patients, and the increase in brain selenium correlated with lack of decline in performance on the Mini-Mental Status Examination (MMSE) (43). Thus, brain selenoproteins, including SELENOP1, may be important for preventing Alzheimer's pathology.

Zn^2+^ metabolism is altered in AD, resulting in abnormally enriched Zn^2+^ environments within the AD brain (44). Zinc-binding sites on the Aβ peptide result in Zn^2+^ mediated aggregation of Aβ and amyloid plaque formation. Furthermore, both the neuroprotective role of SELENOP1 against Aβ toxicity and the role of Zn^2+^ in protecting the cell against oxidative damage, could be working together to reduce the levels of Aβ stress observed in AD pathology, however more studies need to be done to further elucidate their contribution to alleviating oxidative stress.

We did not observe an increase in total brain Zn^2+^ levels with ICP-OES, suggesting only changes in local Zn^2+^ distribution. The absence of SELENOP1 could result in increased oxidative stress in the brain and lead to Zn^2+^ release from MT3 (45), possibly inducing an upregulation of the Zn^2+^ storage protein. In the absence of SELENOP1, we observed a seemingly paradoxical impairment of Zn^2+^ release despite an overall increase in intracellular chelatable Zn^2+^. This was surprising, since most chelatable Zn^2+^ is thought to be localized to synaptic vesicles (46). Overexpression of MT3 in SELENOP1^−/−^ mice may affect the subcellular distribution of Zn^2+^ by limiting the amount of free Zn^2+^ available for loading into the synaptic vesicles. The protein Reelin can increase release of a subset of synaptic vesicles (47). This increase is dependent on Reelin binding to ApoER2. SELENOP1 is another ligand for ApoER2 (48, 49), and thus may also modulate vesicle release, possibly including zincergic vesicles. A decrease in release of Zn^2+^ vesicles could result in a “back-up” of these vesicles, which could contribute to the observed increase in chelatable Zn^2+^. The APOE ε4 allele of the major ligand ApoE for ApoER2, increases risk of Alzheimer's disease (50). Interestingly, the APOE ε4 allele is also associated with decreased selenium in the brain (51), again suggesting a possible role for selenium and selenoproteins in preventing Alzheimer's disease.

We found an interesting sex difference in Zn^2+^ levels in the CA3 region of the hippocampus (Figure 2). Female mice generally had higher Zn^2+^ levels but with some variability. Previous studies have shown higher Zn^2+^ levels in female wild-type mice and AD mouse models (52, 53), although these studies did not agree on the age of sex differences. The differences suggest Zn^2+^ as a possible explanation for the increased risk of AD in women (54). Researchers recently discovered that estrogen increases hippocampal Zn^2+^, which was cycle-dependent in female mice (55, 55), which may explain the increased Zn^2+^ in other studies as well as the variability of the current results.

SELENOP1 contains a putative metal-binding domain that can potentially bind Zn^2+^ with a high affinity. Our results demonstrate that SELENOP1 is capable of binding Zn^2+^ as well as Co^2+^ and Ni^2+^. However, our finding that selenium deficiency and inhibition of GPX4 suggest that the absence of selenium and loss of antioxidant selenoprotein function in the SELENOP1 KO mice is responsible for the increased Zn^2+^ levels. The reason for the presence of the functional metal-binding domain of SELENOP1 remains unknown. It is possible that Zn^2+^ binding could alter the affinity of SELENOP1 for the ApoER2 receptor, allowing for regulation of Se by excess Zn^2+^. Additionally, the homology of the Zn^2+^ binding domain with metalloproteases such as ADAM10 and neprilysin (Figure 1) opens the possibility of an enzymatic role in proteolysis. ADAM10 has a protective role in Alzheimer's disease as a putative α-secretase that promotes non-amyloidogenic cleavage of amyloid precursor protein, preventing amyloid beta formation (56).

The data presented here indicate that SELENOP1 may play a crucial role in the maintenance of brain Zn^2+^. The SELENOP1 gene can affect Zn^2+^ metabolism and synaptic release from neuronal synapses. Zn^2+^ is increased in Alzheimer's disease and interacts with amyloid beta (17), and can also promote tau phosphorylation (18). Thus, Zn^2+^-binding properties of SELENOP1 could contribute to the association of SELENOP1 with amyloid beta plaques in Alzheimer's disease (8). The SELENOP1-Zn^2+^ interaction has potentially important implications in neuronal function and synaptic physiology.

## Data Availability Statement

The original contributions presented in the study are included in the article/supplementary material, further inquiries can be directed to the corresponding authors.

## Ethics Statement

The animal study was reviewed and approved by University of Hawaii IACUC.

## Author Contributions

FB, CS, DT, and AK designed the research. DT, AH, JP, and RR performed the research. FB analyzed the data and wrote the manuscript. All authors contributed to the article and approved the submitted version.

## Conflict of Interest

The authors declare that the research was conducted in the absence of any commercial or financial relationships that could be construed as a potential conflict of interest.
